# The Expression of NP847 and Sox2 after TBI and Its Influence on NSCs

**DOI:** 10.3389/fncel.2016.00282

**Published:** 2016-12-22

**Authors:** Jun Gu, Yifeng Bao, Jian Chen, Chuanjun Huang, Xinghua Zhang, Rui Jiang, Qianqian Liu, Yonghua Liu, Xide Xu, Wei Shi

**Affiliations:** ^1^Department of Neurosurgery, Affiliated Hospital of Nantong UniversityNantong, China; ^2^Department of Neurosurgery, Yancheng Third People's HospitalYancheng, China; ^3^Jiangsu Province Key Laboratory for Inflammation and Molecular Drug Target, Nantong UniversityNantong, China; ^4^Department of Neurosurgery, The First People's Hospital of WujiangSoochow, China; ^5^Department of Anatomy and Neurobiology, Nantong UniversityNantong, China

**Keywords:** neural stem cell, p-nNOS, Sox2, transcription factors, cellular proliferation, mouse model

## Abstract

The proliferation and differentiation of neural stem cells (NSCs) is important for neural regeneration after cerebral injury. Here, for the first time, we show that phosphorylated (p)-ser847-nNOS (NP847), rather than nNOS, may play a major role in NSC proliferation after traumatic brain injury (TBI). Western blot results demonstrated that the expression of NP847 and Sox2 in the hippocampus is up-regulated after TBI, and they both peak 3 days after brain injury. In addition, an immunofluorescence experiment indicated that NP847 and Sox2 partly co-localize in the nuclei of NSCs after TBI. Further immunoprecipitation experiments found that NP847 and Sox2 can directly interact with each other in NSCs. Moreover, in an OGD model of NSCs, NP847 expression is decreased, which is followed by the down-regulation of Sox2. Interestingly, in this study, we did not observe changes in the expression of nNOS in the OGD model. Further research data suggest that the NP847-Sox2 complex may play a major role in NSCs through the Shh/Gli signaling pathway in a CaMKII-dependent manner after brain injury.

## Introduction

Cerebral injury can cause irreversible damage to the brain, as neurons are non-renewable (Griffin, 2003; Zhang et al., 2013). Endogenous neural stem cells (NSCs) proliferate and migrate to sites of brain tissue damage to promote neuroregeneration after a stroke or traumatic brain injury (TBI) (Chen et al., 2003; Thored et al., 2006, 2007). However, due to their limited number, endogenous NSCs are insufficient for neuronal regeneration (Vazey et al., 2006; Delcroix et al., 2010). Therefore, to enhance the survival and proliferation abilities of endogenous NSCs as much as possible for neuronal regeneration, it is critical to investigate the mechanism of endogenous NSC mobilization after brain injury.

Numerous studies confirm that nNOS appears in different brain regions (Di Girolamo et al., 2003; Jinno and Kosaka, 2004; Endo et al., 2016), and it plays a critical role in central nervous system (CNS) injury. In TBI, nNOS is closely related to secondary brain damage by peroxide nitrite (Adak et al., 2000), and in brain ischemia, the excessive activation of nNOS will increase damage to the CNS (Hsu et al., 2014; Wu et al., 2014). Recently, Luo et al. reported that NSC-derived nNOS is essential for neurogenesis, while neuron-derived nNOS exerts negative control on neurogenesis (Luo et al., 2010). We intended to study the role of nNOS in NSC mobilization after TBI; however, our experimental results indicated that nNOS is not a key factor in this process. Interestingly, we found that the expression of NP847, which is phosphorylated (p)-ser847-nNOS, in the hippocampus peaked at an early stage after TBI. It is known that nNOS activity can be enhanced by phosphorylation at ser1412 (Osuka et al., 2013) and inhibited by phosphorylation at ser847 (Wang et al., 2010). Therefore, we focused on NP847 to further study its role in the neuroprotection of NSC mobilization after TBI.

It has been reported that NP847 can protect neurons against neurotoxicity by decreasing the activity of nNOS (Nakane et al., 1991; Bredt et al., 1992). The associated decrease in enzyme activity is thought to be partially attributable to the suppression of CaM binding to the enzyme (Komeima et al., 2000). However, few studies have revealed the role of NP847 in the mobilization of NSCs for neuroregeneration after brain injury. Our previous experiments first showed that NP847 is expressed in the DG region of the hippocampus, which is the region that has the most abundant NSCs (Gould et al., 1999; Deng et al., 2010), and NP847 appears in the nuclei of NSCs. However, the biological behavior of NP847 in the mobilization of NSCs and its mechanism are not clear. Here, we focus on the relationship between the mobilization of NSCs and the activation of NP847 after TBI and further investigate the molecular mechanisms of the signaling pathways involved in the self-renewal and proliferation of NSCs.

Sox2 belongs to the B1 subgroup of the Sox (sry-related HMG box-containing) family group, and it is a central molecule that is closely associated with stem cells (Liu et al., 2013). Our previous experiments first showed that NP847 and Sox2 demonstrate a similar trend toward variation in the DG region of the hippocampus, which indicates that they have a certain relationship with NSCs. Moreover, as a nuclear transcription factor, Sox2 can regulate the transcription of downstream genes. Early research showed that sonic hedgehog (Shh) is a direct target gene of Sox2 (Zhang et al., 2016). Furthermore, it is a powerful regulator of adult hippocampus neurogenesis and is essential for the maintenance of adult stem cell properties (Cheng et al., 2015). Here, we report that nNOS is phosphorylated at ser847 in NSCs after TBI and that the activated NP847 can interact with Sox2, thereby affecting the expression of Shh to influence the proliferation and self-renewal of NSCs through the Shh/Gli signaling pathway.

## Materials and methods

### Animals and the TBI model

Experiments were performed in accordance with the National Institutes of Health Guidelines for the Care and Use of Laboratory Animals (Bayne, 1996, USA) and were approved by the Chinese National Committee for the Use of Experimental Animals for Medical Purposes, Jiangsu Branch. Male Sprague Dawley rats (*n* = 46) with an average body weight of 250 g (range 220–275 g) were used in this study. A TBI model described previously was used with modifications (Logan et al., 1992; Shi et al., 2012). After deeply anesthetizing the rats with chloral hydrate (10% solution), the heads of the rats were fixed in a stereotactic frame, and under aseptic conditions, a micro-knife was inserted into the right cortex,3 mm lateral, parallel from the midline, with an antero-posterior surgical incision (5 mm long, 3 mm deep, and 1 mm wide). Thereafter, the scalps were sutured. Sham-controlled rats were subjected to identical procedures as the experimental rats, except for the insertion of a micro-knife into the brain. After all the procedures, the animals were returned to their cages and allowed free access to food and water. Animals were housed under a 12-h light/dark cycle, and the room temperature (RT) was kept at 20 ± 2°C. Experimental animals (*n* = 3 per time point) were sacrificed to extract proteins for western blot analysis at 12 h, 1, 3, 5, 7, 14, and 28 days after injury. Sham rats were sacrificed on the third day. Additional experimental animals for sections were sacrificed at 3 days for the sham group and at 3 days after TBI. Experimental animals (*n* = 3 per time point) were sacrificed at 1, 3, and 5 days for immunoprecipitation. Sham rats were sacrificed on the third day. Four rats were lost in the TBI group. All efforts were made to minimize the number of animals used and the suffering of the animals.

### Cell culture

Embryonic NSCs were isolated from embryonic day 16 (E16) rat cortex as previously described (Pollard et al., 2006). Cells were maintained in floating culture in proliferation medium containing 20 ng/ml basic fibroblast growth factor (bFGF, Abcam), 20 ng/ml epidermal growth factor (EGF, Abcam), and 2% B27 supplement (Abcam) and were passaged every 4–6 days. These embryonic NSCs still proliferated and had a self-renewal capacity. The cells were able to generate differentiated progeny until the 10th passage. Embryonic NSCs from the 2nd to the 10th passages were used in this study. Adult NSCs were isolated and cultured as previously described, with some modifications. In brief, the dentate gyri of 2-month-old female mice (three mice were used in each primary isolation experiment) were dissected and digested with 0.125% trypsin (Gibco) and 250 U/ml DNase I (Sigma-Aldrich) at 37°C for 20 min, and the undifferentiated progenitors were enriched by centrifugation with Percoll.

Adult NSCs were maintained in floating culture in proliferation medium similar to that of embryonic NSCs. The proliferation, self-renewal capacity, and multiple differentiating potential of adult NSCs were as easily identified as for the embryonic NSCs. Adult NSCs of the second to fourth passages were used in this study.

For lentivirus transfection and cell co-culture experiments, embryonic or adult NSCs were plated on coverslips (2 cm^2^) coated with polylysine at a cell density of 1 × 10^4^ cells/cm^2^ and were cultured as a monolayer.

Primary hippocampal neurons were isolated from an embryonic day 18 (E18) mouse and cultured in neurobasal medium (Gibco) containing 2% B27 supplement, as reported. Cultured neurons were identified after 10 days *in vitro*, and the proportion of β-III-tubulin^+^ cells was approximately 92%.

### Co-culture of NSCs and neurons

To simulate the pathological process of NSC mobilization after TBI, NSCs were co-cultured with neurons stimulated by glutamate (gNeurons) *in vitro*. Before co-culture, NSCs were plated on polylysine coverslips and cultured for 24 h. Then, the coverslips with NSCs were turned over and placed on hippocampal neurons grown in dishes for 10 days to undergo co-culture for 24 h. Neurobasal medium containing 20 ng/ml bFGF, 20 ng/ml EGF, and 2% B27 was used as the co-culture medium. After co-culture, the upper coverslip with the NSCs was removed from the dish with neurons to perform immunecytochemistry on the NSCs.

All cultures, including NSCs, neurons, and co-cultures, were maintained in an incubator with a humidified atmosphere of 95% air and 5% CO_2_ at 37°C.

### Western blot analysis

After the administration of an overdose of chloral hydrate, the rats were sacrificed at different time points postoperatively. The cortex tissue surrounding the wound (extending 3 mm from the incision) as well as equal parts of the normal and the contralateral cortex were dissected and stored at−80°C until use. To prepare the lysates, frozen cortex tissue samples were weighed and minced with eye scissors on ice. Samples were homogenized in lysis buffer (1% sodium dodecyl sulfate (SDS), 1% Triton X-100, 50 mmol/L Tris, 1% NP-40, pH 7.5, 5 mmol/L EDTA, 1% sodium deoxycholate, 1 mg/ml leupeptin, 10 mg/ml aprotinin, and 1 mmol/l PMSF), centrifuged at 13,000 rpm, and 4°C for 20 min, and the supernatant fractions were collected. The cell cultures were lysed with sodium lauryl sulfate loading buffer and stored at −80°C until use for immune blot analysis. After determining the protein concentrations with a Bradford assay (Bio-Rad), the samples were subjected to SDS-polyacrylamide gel electrophoresis (SDS-PAGE), followed by transfer to a polyvinylidine difluoride filter (PVDF) membrane. The membrane was blocked with 5% skim milk for 2 h and incubated with primary antibodies against nNOS (anti-rabbit, 1:1000, Abcam), Sox2 (anti-rabbit, 1:1000, Abcam), NP847 (anti-rabbit/mouse, 1:1000, Abcam), shh (anti-mouse, 1:1000, Santa Cruz), and GAPDH (anti-rabbit, 1:1000, Santa Cruz) at 4°C overnight. Subsequently, the membrane was incubated with a goat-anti-mouse or goat-anti-rabbit-conjugated horseradish peroxidase secondary antibody (1:10,000, Southern Biotech) for 2 h, and the proteins were visualized using an enhanced chemiluminescence system (ECL; Pierce Company, USA).

### Immunofluorescence

Sections were first blocked with a 10% normal serum-blocking solution of the same species as the secondary antibody containing 3% (w/v) BSA, 0.1% Triton X-100, and 0.05% Tween-20 for 2 h at RT to avoid nonspecific staining. Then, the sections were incubated with the following primary antibodies overnight at 4°C:anti-NP847 antibody (anti-rabbit/mouse, 1:100, Abcam), anti-Sox2 antibody (anti-rabbit, 1:100, Abcam), anti-Nestin mouse/rabbit monoclonal primary antibodies (anti-mouse, a marker of NSCs, 1:100, Abcam), anti-shh antibody (anti-mouse, 1:200, Santa Cruz Biotechnology), anti-CaMKII antibody (anti-mouse, 1:100, Abcam), anti-Ki67 mouse/rabbit polyclonal antibody (a marker of proliferation, 1:100; Abcam) and anti-mouse/rabbit PCNA (Santa Cruz Biotechnology; 1:100). Then, sections were treated with a mixture of FITC—and TRITC-conjugated secondary antibodies for 2 h at 4°C. To detect the morphology of apoptotic cells, sections were stained with DAPI (0.1 mg/ml in PBS; Sigma) for 40 min at 30°C. The stained sections were examined with a Leica fluorescence microscope (Leica, DM 5000B; Leica CTR 5000; Leica, Germany)

### Statistical analysis

All values were expressed as the mean ± SEM. The data were compared using Student's *t*-test. *P* < 0.05 was considered statistically significant. Each experiment had at least three replicates per condition. The SEM refers to the standard error of the mean.

## Results

### The expression of NNOS/NP847/Sox2 in the hippocampus after brain injury

**Figure 1:**
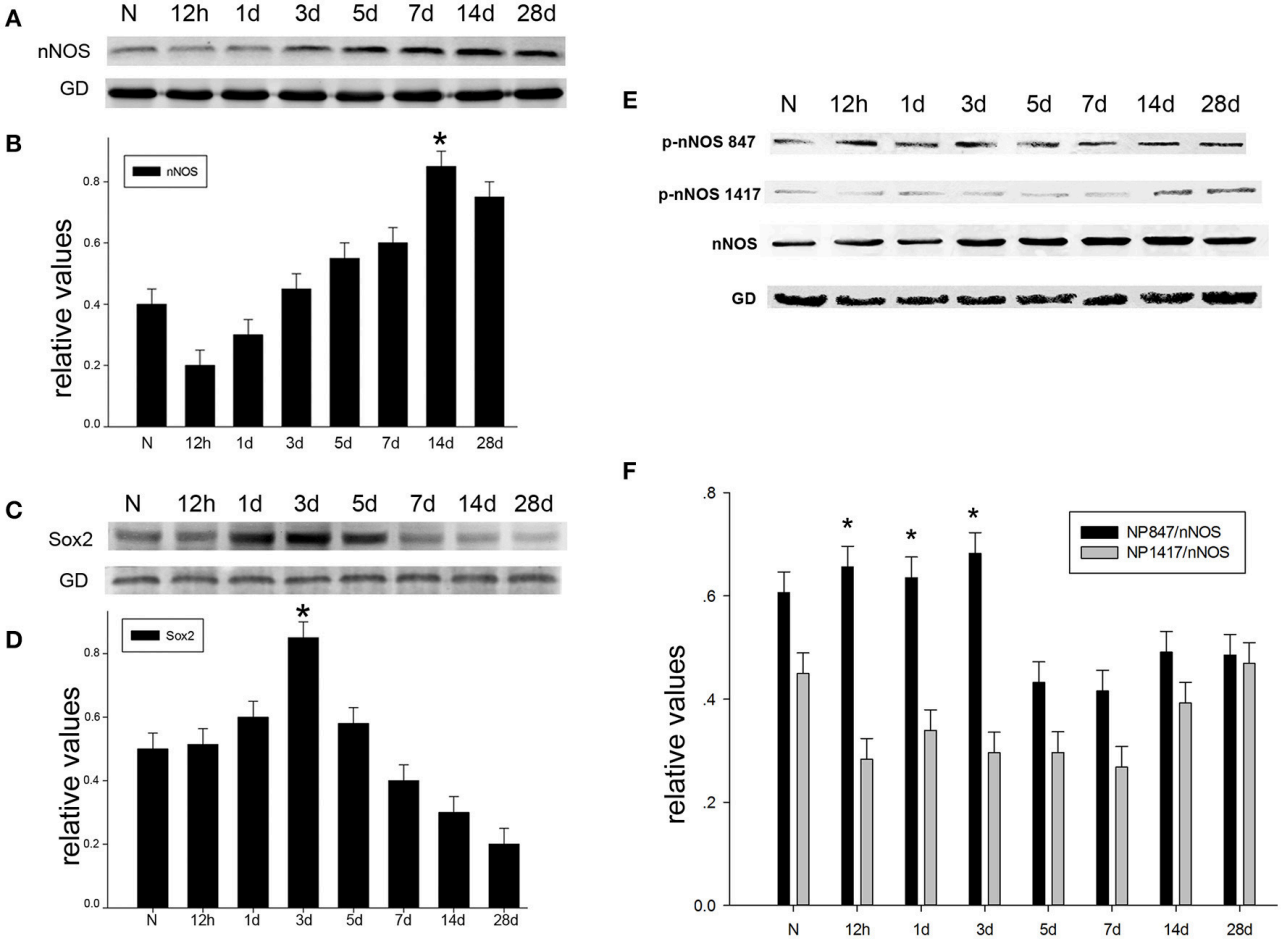
**The time-dependent variation in nNOS/Sox2/p-nNOS protein expression after brain injury in rats. (A)** The time course of the protein expression of nNOS in adult rat hippocampus following TBI by Western blot. **(B)** The relative protein contents of nNOS were calculated by densitometry analysis, and the data were normalized to corresponding GAPDH levels. **(C)** The time course of Sox2 protein expression in adult rat hippocampus following TBI as determined by Western blot. **(D)** The relative protein contents of Sox2 were calculated by densitometry analysis, and the data were normalized to corresponding GAPDH levels. **(E)** The time course of the protein expression of p-nNOS in adult rat hippocampus following TBI as determined by Western blot. **(F)** Graphs (relative optical density) quantifying the intensity of the staining of p-nNOS compared to corresponding nNOS levels at each time point. The values are presented as the mean ± SEM (*n* = 3). ^*^P < 0.05 indicates significant differences compared to the sham group.

**Figure 2:**
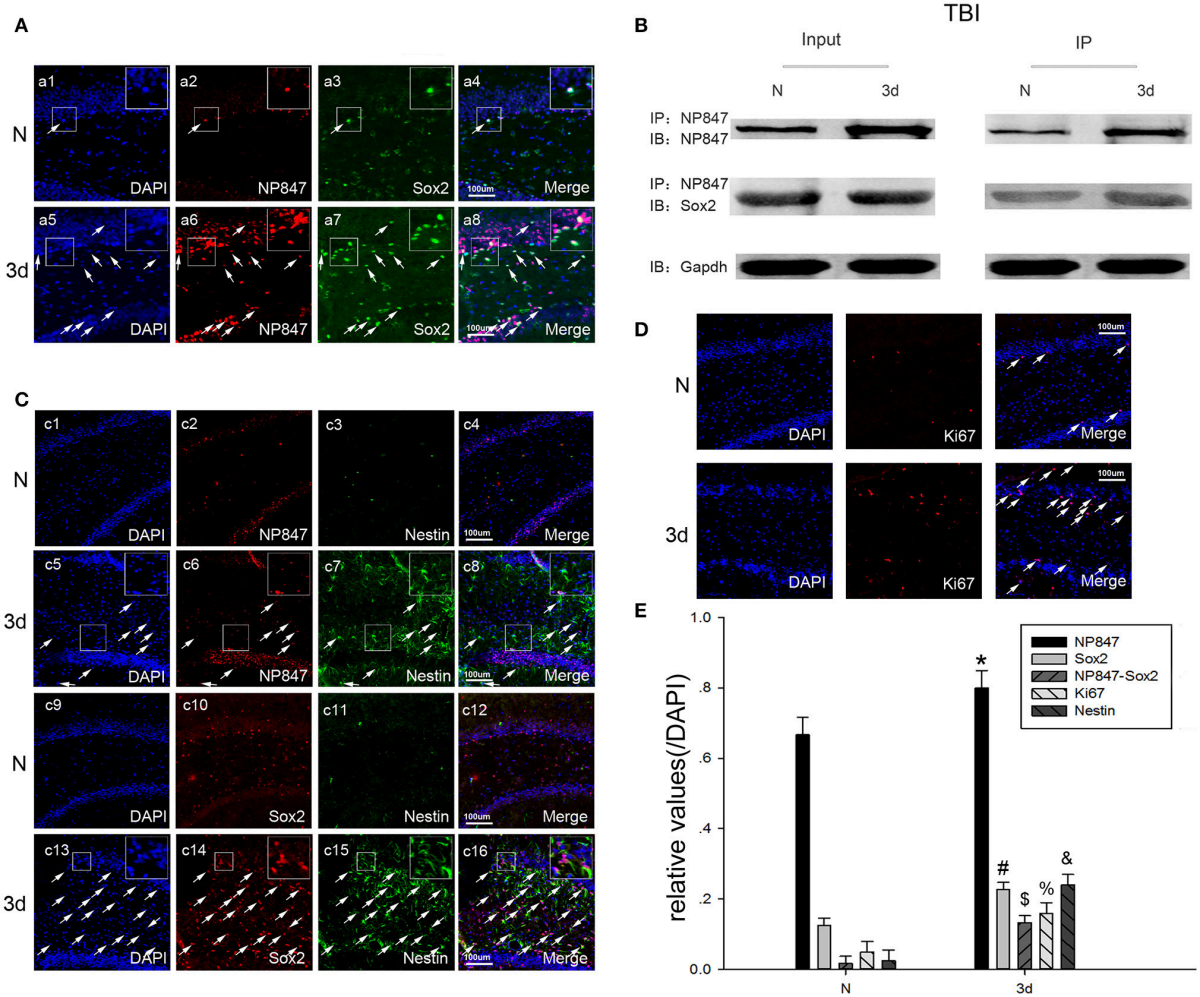
**The expression of NP847 and Sox2 in the brain hippocampus. (A)** In the adult rat brain hippocampus at 3 days after TBI, horizontal sections were labeled forNP847 (red), Sox2 (green), and the nuclear marker DAPI (blue). The white color visualized in merged images represents the co-localization of NP847 and Sox2 (arrow). **(B)** Immunoprecipitation showed that the interaction of NP847 and Sox2 significantly improved in the experimental group 3 days after TBI compared to the sham group. **(C)** Double immunofluorescence staining for Nestin (green, c1–8) and NP847 (red, c1–8). The arrows show the co-localization of NP847 and Nestin (c8). Double immunofluorescence staining for Nestin (green, c9–16) and Sox2 (red, c9–16). The arrows show the co-localization of Sox2 and Nestin (c16). **(D)** Immunofluorescence staining for DAPI (blue) and Ki67 (red). The arrows show the co-localization of Ki67 and DAPI. **(E)** Statistical results for the expression of NP847, Sox2, NP847-Sox2, Nestin and Ki67 in the hippocampus 3 days after brain injury compared with the sham group. ^*#$%&^*P* < 0.05 indicates significant differences compared to the sham group. Scale bars: 100 μm.

To probe the possible function of nNOS, Sox2, and NP847 in brain injury, a TBI model was established in adult rats. We used western blotting analysis to investigate the temporal patterns of the expression of nNOS/NP847/Sox2 in the brain hippocampus after brain injury. The level of nNOS was relatively low in normal hippocampus and then progressively increased starting at12 h after TBI, peaked at day 14 (*P* < 0.05), and then gradually decreased to normal levels (Figures 1A,B). Sox2 expression gradually increased starting at 12 h following TBI and peaked at day 3 compared with the normal group (Figures 1C,D). NP847, rather than NP1417, demonstrated an identical trend to Sox2 (Figures 1E,F), indicating that NP847 might have a connection with Sox2. To further identify the variation and distribution of NP847 and Sox2 after TBI, immunofluorescent labeling analysis was performed on sections 3 days following TBI and showed relatively high NP847- and Sox2-positive staining compared with the sham operated group, which was consistent with the western blotting data (Figures 2A,E). The expression trends of nNOS and NP847 were not synchronized, which demonstrates that they have different functions. After brain injury, the expression of NP847 peaked earlier than that of nNOS, indicating that NP847 might have a more vital impact than nNOS during the early stage of neural despair after brain injury.

### NP847 directly interacts with Sox2

Immunofluorescent labeling showed that NP847 and Sox2 co-localized in the DG region of the hippocampus (Figure 2A), so we hypothesized that Sox2 is a potential binding partner for NP847. To validate this possible interaction, an immunoprecipitation assay was designed. The assay demonstrated that NP847 could interact with Sox2 both in normal brain tissues and in the brain after injury, and the interaction was enhanced after injury (Figure 2B).

### The expression and distribution of NP847 and Sox2 in the hippocampus after TBI

NP847 partly co-localized with Nestin in the DG region of the hippocampus, similar to Sox2 (Figures 2C,E). Statistical results showed that 3 days after brain cortex injury, the expression of NP847, Sox2, NP847-Sox2, Nestin, and Ki67 in the hippocampus increased compared to the sham group (Figures 2D,E). This indicated that NP847 and Sox2 in the hippocampus might have a connection with the autologous mobilization of NSCs.

### The expression and distribution of NP847 and Sox2 in neurospheres cultured with damaged neurons

**Figure 3:**
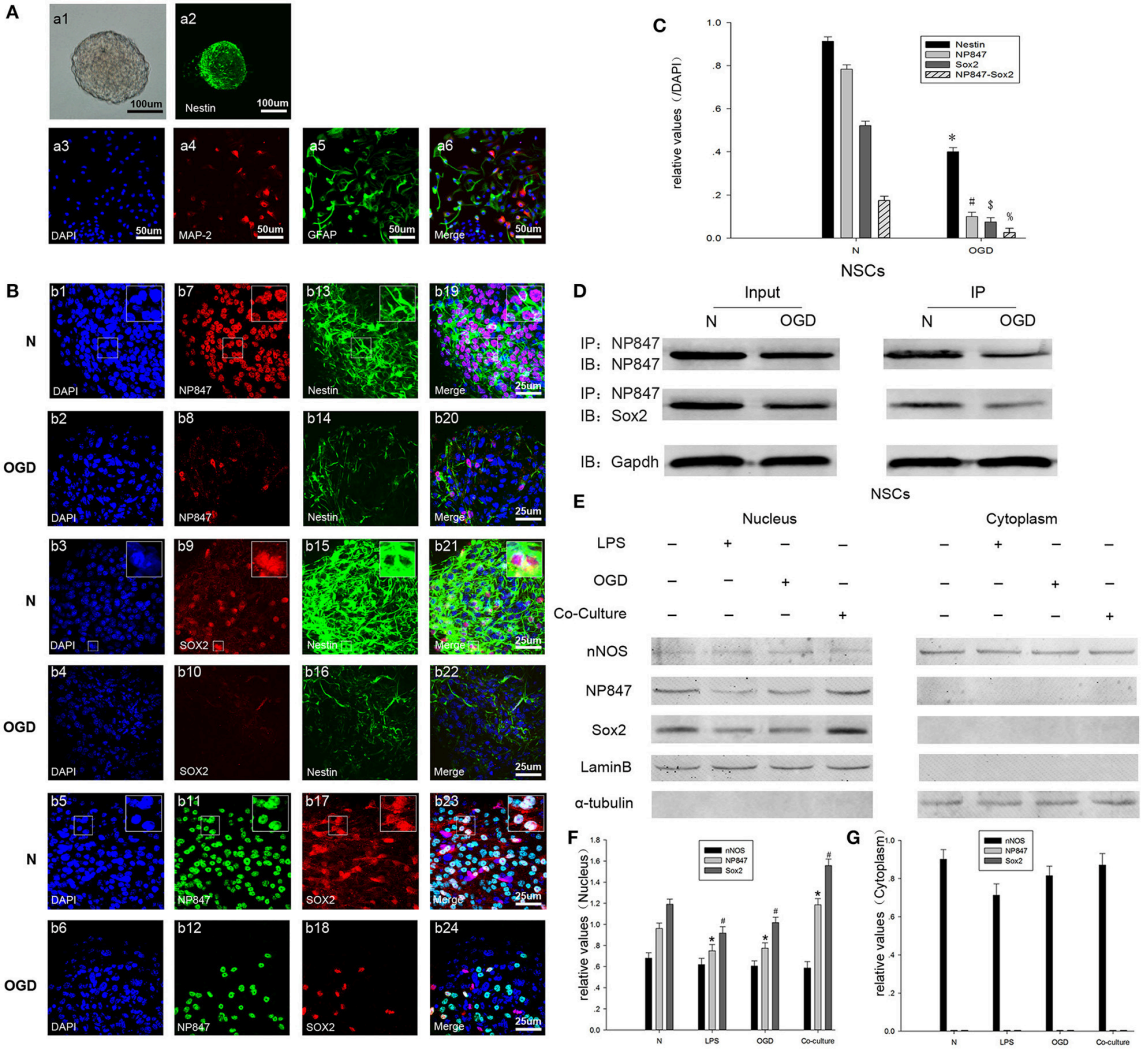
**NP847 co-localized with Sox2 in the nuclei of NSCs. (A)** Neurospheres were cultured as low-density cell suspensions in the presence of epidermal growth factor and basic fibroblast growth factor. And neurosphere was observed by light microscope (a1, scale bar: 100 μm). Then neurospheres were planted on coverslips and the expression of Nestin was detected (a2, scale bar: 100 μm). Fetal bovine serum was added into the NSCs culture medium to stimulate the differentiation of NSCs and expression of MAP-2 (a4) and GFAP (a5) was detected. Scale bars: 50 μm. **(B)** Immunofluorescence was performed to detect NP847 (red, b7, b8; green, b11, b12), Sox2 (red, b9, b10, b17, b18), and Nestin (green, b13–16). The nuclei were stained with DAPI (blue, b1–6). (b19) and (b20) show the co-localization of NP847 (red) and Nestin (green) (small panels represent high magnification images). (b21) and (b22) show the co-localization of Sox2 (red) and Nestin (green) (small panels represent high magnification images). In (b23) and (b24), the white color visualized in merged images represents the co-localization of NP847 and Sox2 in the nuclei of NSCs (small panels represent high magnification images). The expression of NP847, Sox2, and Nestin decreased after OGD (oxygen glucose deprivation) compared with the normal group. Scale bars: 25 μm. **(C)** Graphs (relative optical density) quantifying the intensity of **(B)**. **(D)** Immunoprecipitation showed that the interaction of NP847 and Sox2 significantly decreased in NSCs suffered from OGD compared to the sham group. **(F)** To further determine the sub cellular locations of NP847 and Sox2 in NSCs, primary NSCs were treated with either LPS (100 μM) or OGD for 12 h. Then, nuclear extracts and cytoplasmic extracts were prepared, and protein expression was examined by western blotting. Neuronal NOS is mostly located in the cytoplasm of NSCs, while NP847 and Sox2 are confined to the nuclei of NSCs. Panels **(F)** and **(G)** are graphs (relative optical density) quantifying the intensity of **(E)**. The values are presented as the mean ± SEM (*n* = 3). ^%$#*^P < 0.05 indicates significant differences compared to the sham group.

The loss and death of neurons are the major contributors to nerve injury after TBI; therefore, we set up a co-culture model with NSCs isolated from embryonic day 16 (E16) rat cortex and neurons stimulated by glutamate to simulate the process of NSCs mobilization after TBI. And our NSCs isolated from embryonic rat cortex are valuable which is characterized by immunostaining and differentiation assay (by adding fetal bovine serum into the NSCs culture medium) (Figure 3A). The expression of NP847 and Sox2 in the NSCs increased and was induced by damaged neurons, whereas the expression of nNOS expression did not apparently change (Figure 3E).

### The expression and distribution of NP847 and Sox2 in neurospheres in the NSC OGD model

To further study the mechanism by which NP847/Sox2 affects the renewal of NSCs, we established an OGD model. Then, protein expression patterns were examined in whole neurosphere cultures using confocal microscopy. As a marker of NSCs, Nestin expression in the neurosphere obviously decreased in the OGD model compared with the normal state, which indicated that the quantity of active NSCs decreased (Figures 3B,C). Neurosphere sections showed strong immune reactivity for both NP847 and Sox2 in normal NSCs, and they partly co-localized with each other (Figure 3B, row 5). The co-localization of immune reactivity for NP847 and Sox2 in the neurosphere suggests that these two proteins may interact with each other in NSCs, and this was further verified by an IP assay (Figure 3D). With immune fluorescent labeling, we found that NP847 and Sox2 were confined to the nucleus in neurospheres and nNOS was mostly located in the cytoplasm (data not shown). Furthermore, we confirmed whether OGD or LPS induced the nuclear transportation of nNOS/NP847/Sox2 using Western blotting, and our results were consistent with the immunofluorescence results showing that NP847/Sox2 was located in the nucleus and nNOS was mostly in the cytoplasm (Figures 3E–G).

### The NP847-Sox2 complex modulates the Shh signaling pathway in NSCs

**Figure 4:**
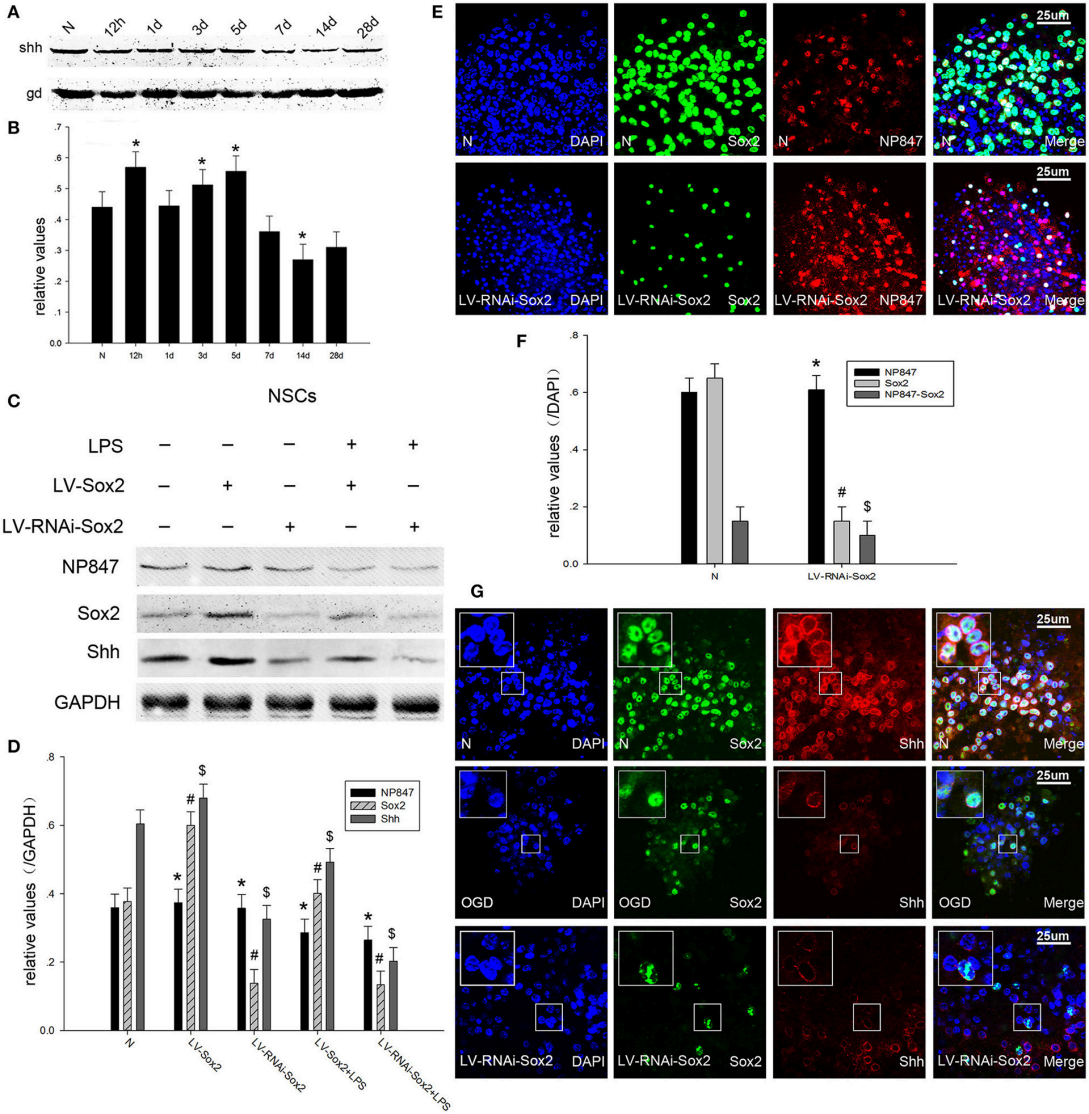
**NP847 facilitates Sox2-modulated Shh transcription. (A)** The time course of Shh protein expression in adult rat hippocampus following TBI as determined by Western blot. **(B)** The relative protein contents of Shh were calculated by densitometry analysis, and the data were normalized to corresponding GAPDH levels. **(C)** Primary NSCs were treated with LPS (100 μM) or transfected with LV-Sox2 or LV-RNAi-Sox2. Then, cell extracts were prepared and protein expression was examined by western blotting. Panel **(D)** is the histogram (relative optical density) quantifying the intensity of **(C)**. **(E)** Immunofluorescence was performed to detect the expression of Sox2 and NP847 in normal NSCs and NSCs infected by LV-RNAi-Sox2. **(F)** Statistical results for the expression of NP847, Sox2 and NP847-Sox2 in normal NSCs and NSCs infected by LV-RNAi-Sox2. **(G)** Immunofluorescence was performed to detect the expression of Sox2 and Shh in normal NSCs (up line), NSCs suffering under OGD treatment (middle line) and NSCs infected by LV-RNAi-Sox2 (bellow line) (small panels represent high magnification images). Scale bars: 25 μm. ^*#$^*P* < 0.05 indicates significant differences compared to the sham group.

It was reported that Shh is a direct target gene of Sox2 and is up-regulated after ischemia, as it can modulate adult hippocampal neurogenesis. Because of this, we investigated whether the NP847-Sox2 complex could modulate downstream Shh transcriptional activity after brain injury. Our results showed that Shh was gradually increased 12 h following TBI and decreased after 5 days compared with the normal group (Figures 4A,B). Neurosphere immunofluorescence indicated that Shh was located in the nuclear membrane of NSCs (Figure 4G) and its expression decreased after OGD, which was consistent with the western blot results (Figures 4C,D). When LV-Sox2 was transfected into NSCs, we found that Sox2 and Shh increased compared with normal NSCs. In comparison, when LV-RNAi-Sox2 was transfected into NSCs, we found that Sox2 and Shh decreased in comparison with normal NSCs, while the expression of NP847 did not remarkably change (Figures 4E,F). And the expression of NP847 in NSCs suffered from LPS descent compared with normal NSCs (Figures 4C,D).

### Camkii phosphorylates nNOS at ser847 and promotes NSC proliferation

**Figure 5:**
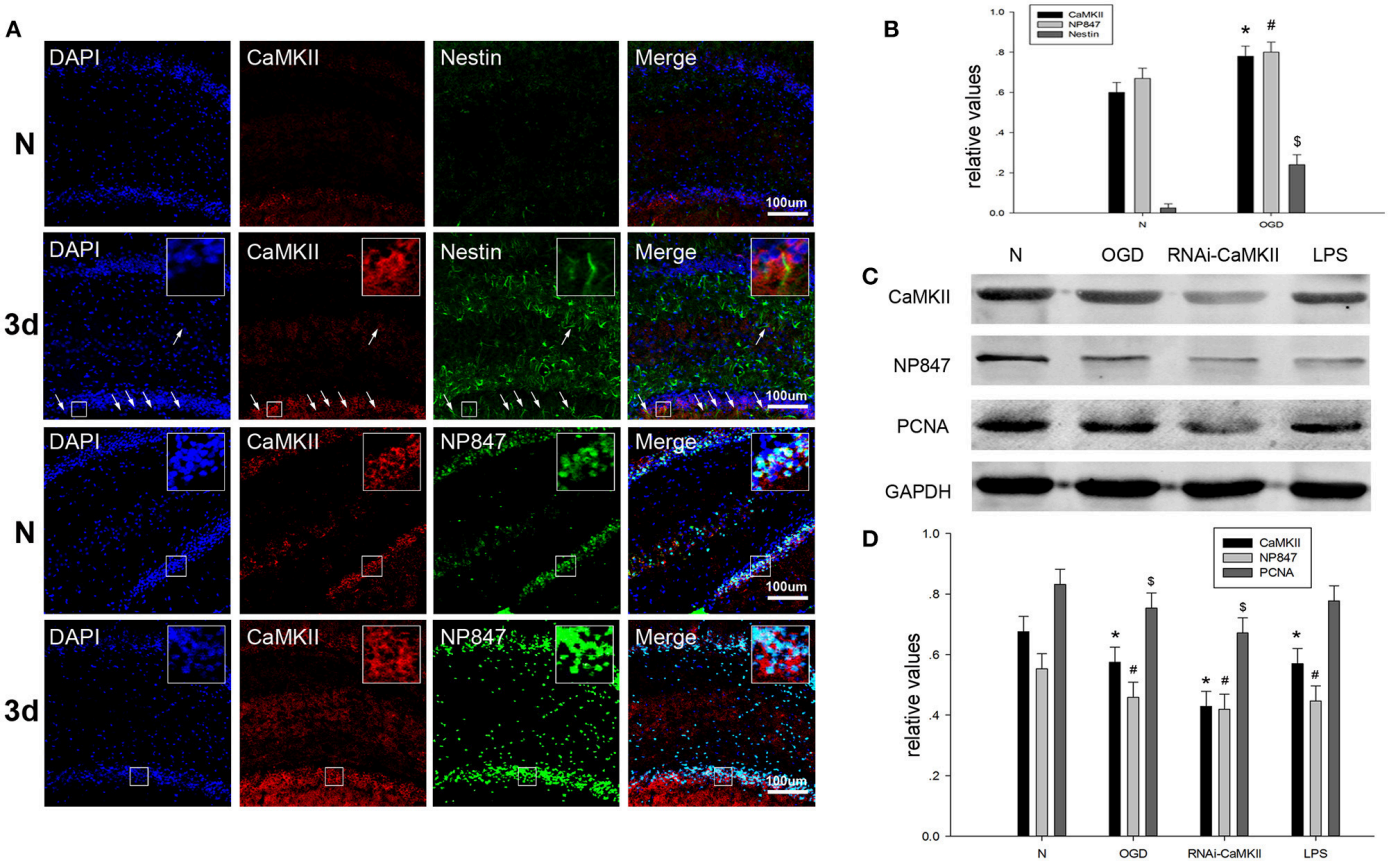
**CaMKII enhances the expression of NP847 in NSCs. (A)** Horizontal sections of adult rat brain hippocampus 3 days after TBI were labeled fo rCaMKII (red), Nestin (green, first and second line) and NP847 (green, third and fourth line) (small panels represent high magnification images). Scale bars: 100 μm. **(B)** Graphs (relative optical density) quantifying the intensity of **(A)**. **(C)** Primary NSCs were treated with LPS (100 μM) or OGD for 12 h or transfected by LV-RNAi-CaMKII. Then, cell extracts were prepared, and protein expression was examined by western blotting. **(D)** Graphs (relative optical density) quantifying the intensity of **(C)**. The values are presented as the mean ± SEM (*n* = 3). ^#*$^*P* < 0.05 indicates significant differences compared to the sham group.

Neuronal nitric oxide synthase is phosphorylated at ser847 by CaMKII, which is activated by phosphorylation at Thr286. We assessed whether brain injury affects the amount of CaMKII in NSCs. Brain tissue sections showed that CaMKII demonstrated enhanced co-localization with Nestin in the hippocampus after brain injury compared to the sham group (Figures 5A,B). The western blot results showed that the expression of CaMKII in NSCs suffering from treatment under OGD conditions and LPS decreased compared to normal NSCs (Figures 5C,D). To further validate whether CaMKII phosphorylated nNOS at ser847, LV-RNAi-CaMKII was transfected into NSCs, and we found that the protein levels of CaMKII, NP847, Sox2, and PCNA decreased compared with normal NSCs, which indicates that NP847 positively impacted the proliferation of NSCs (Figures 5C,D).

The above results suggest that, in brain injury, the increased activity of CaMKII leads to the increased phosphorylation of nNOS at Ser847 under basal conditions, resulting in the enhanced interaction of NP847 and Sox2 to facilitate the activity of NSCs through the Shh signaling pathway.

### The formation of the NP847-Sox2 complex enhances the self-renewal of NSCs in a NSC/gNeuron co-culture model

**Figure 6:**
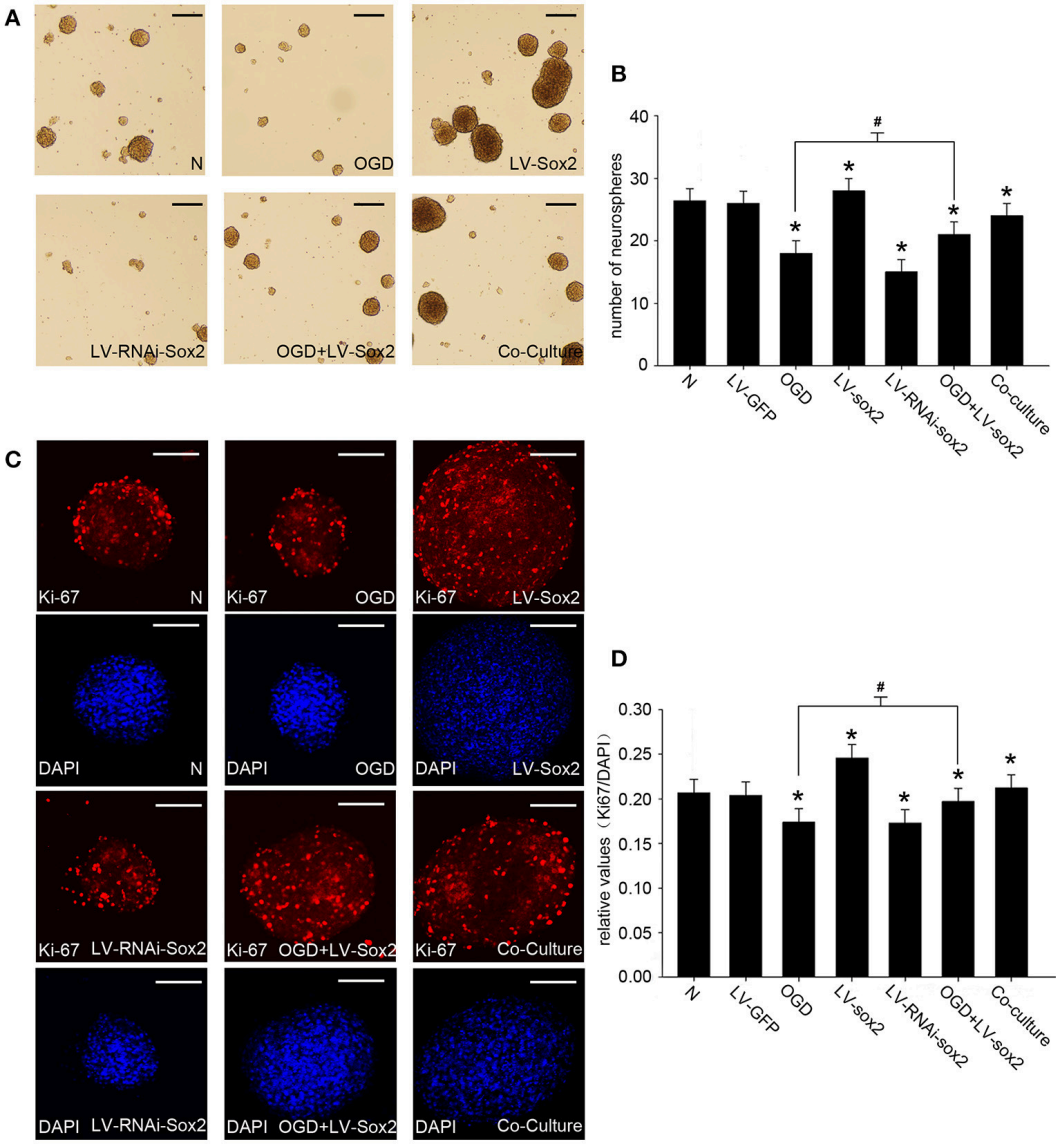
**The expression of Sox2 modulates the self-renewal of NSCs. (A)** Neurospheres were treated by the different methods mentioned above, and the amounts and diameters of the neurospheres were observed by light microscope. Scale bars: 50 μm. **(B)** The number of neurospheres was measured. The values represent the mean ± *SE* (vertical bars) of six independent experiments. **(C)** The amount of Ki67-positive cells was observed with a confocal microscope. Scale bars: 50 μm. **(D)** Quantitative analysis of Ki67-positive cells compared with DAPI. ^*^Indicates a significant difference at *P* < 0.05 compared with the normal group, and ^#^indicates a significant difference at *P* < 0.05 compared with the OGD group. Error bars represent the SEM.

To confirm the effects of Sox2 on the self-renewal of neural progenitor/stem cells, we performed cell proliferation assays by measuring the number of neurospheres (Figures 6A,B) and the amount of Ki67-positive cells (Figures 6C,D). LV-Sox2 treatment significantly increased the ratio of Ki67-positive NSCs and the number of neurospheres. LV-RNAi-Sox2 and OGD treatment reduced the ratio of Ki67-positiveNSCs and the number of neurospheres. The co-culture of neurons stimulated by LPS and NSCs increased the ratio of Ki67-positive NSCs and the number of neurospheres. These results indicate that the NP847-Sox2 association may enhance the self-renewal of neural progenitors/stem cells.

## Discussion

There have been many reports concerning the physiological and pathological changes after brain injury (Kraus et al., 2007; Roth et al., 2014; Kondo et al., 2015; Delbary-Gossart et al., 2016), and the proliferation and migration of autologous NSCs is a focus of study (Hu et al., 2014; Wang et al., 2016). It has been reported that modeling strokes and TBI in animals has allowed the identification of a unique response in the sub ventricular zone (SVZ), where endogenous neural stem/progenitor cells (NSPCs) undergo transient expansion and begin to migrate to sites of tissue damage (Chen et al., 2003; Thored et al., 2006, 2007). This enhanced neurogenic response provides a potential source of multipotent cells capable of integrating into existing neural networks to promote functional recovery (Arvidsson et al., 2002; Lai et al., 2008; Liu et al., 2009). It is universally acknowledged that neurogenesis is easily triggered in brain injuries, such as trauma, stroke and aspiration lesions (Parent, 2003), and that these injuries initiate, activate, and enhance the ability of NSCs to generate neurons in the hippocampus (Imitola et al., 2004).

The observation that nNOS is expressed in a variety of different tissues during different biological processes, especially neurotoxicity, suggests that this protein may be involved in NSC-fate mechanisms (Brito et al., 2010; Jiang et al., 2014). It has been reported that nNOS is a negative regulator of cell proliferation through the generation of NO in the neurogenic regions of the adult mammalian brain (Packer et al., 2003; Moreno-López et al., 2004). Although there are many reports regarding the function of nNOS in neurons and astrocytes (Shin, 2001; Yuan et al., 2004; Eugenin et al., 2007), research investigating on its effects on the proliferation of NSC apoptosis is rare. Thus, we investigated what kind of role nNOS plays in the proliferation of NSCs after brain damage. Unexpectedly and interestingly, our results showed that NP847, but not nNOS, may play a leading role in the mobilization of NSCs for neuroregeneration in the early stages after TBI.

Bredt et al. reported that nNOS is phosphorylated by protein kinases, including calcium-calmodulin-dependent protein kinases (CaMKs) (Bredt et al., 1992). CaMKIIα can phosphorylate nNOS at Ser847 or Ser1412, and NP847 is the product of the phosphorylation of nNOS at the ser847 site (Hayashi et al., 1999; Komeima et al., 2000). It has been reported that NP847 can inhibit the negative regulation of nNOS during neurogenesis by decreasing the activity of nNOS (Osuka et al., 2007). Makino et al. reported that the activation of NP847 in the hippocampus alleviates ischemic insults immediately after SAH to exert a neuroprotective effect (Makino et al., 2015). Moreover, protein phosphatase 2A (PP2A) can dephosphorylate the Ser847 residue of NP847 to recover nNOS enzymatic activity (Komeima et al., 2000; Komeima and Watanabe, 2001). All of these studies suggest that NP847 has a positive effect on the CNS; however, the specific mechanism is still unclear.

Sox2 is pivotal for early development and the maintenance of undifferentiated ESCs (embryonic stem cells). There are many reports showing that Sox2 is expressed in functionally-defined NSCs and maintains their properties (Suh et al., 2007; Andreu-Agullo et al., 2012; Lee et al., 2014). Previous reports have suggested that Sox2 may be a marker of NSCs and their precursors (Zappone et al., 2000). It has been reported that Sox2 deletion causes hippocampal defects with NSCs loss and that the *shh* gene is a direct target of Sox2 (Favaro et al., 2009). However, the role of Sox2 and the relationship between Sox2 and NP847 in the pathological changes involved in the mobilization of NSCs after TBI require further investigation.

In this study, we used a model of TBI to analyze the changes in the mobilization of NSCs and the relative expression of Sox2 after brain injury. Here, we first reported that the expression of NP847 and Sox2 increased in the rat hippocampus after TBI, as indicated by Western blot analysis and immunohistochemistry. Both peaked 3 days after brain injury, which was not consistent with the changes in the expression of nNOS after TBI. Moreover, our immunofluorescence results also showed that NP847 and Sox2 partly co-localized.

Then, we explored the relationship between the two molecules by performing co-immunoprecipitation, and the results showed that the proteins demonstrated an enhanced interaction with each other in the hippocampus of rats suffering from TBI compared with normal hippocampus. These results implied that Sox2 and NP847, not nNOS, may actively affect the mobilization of NSCs in the early stage of brain injury. To further confirm these results, the expression of NP847 and Sox2 in NSCs from E16 rat hippocampus was detected, and immunofluorescence experiments showed they partly co-localized in the nuclei of NSCs. To simulate the pathological process of NSC mobilization after TBI, NSCs were co-cultured with neurons stimulated by glutamate *in vitro*. In this double co-culture model, we also found that NSC proliferation was enhanced and the expression of NP847 and Sox2 was up-regulated after this process.

After TBI, brain tissue surround the damage zone may be showing a hypoxic-ischemic state. Moreover, in an OGD model of NSCs, we observed the down-regulation of NP847 and Sox2, which was consistent with the decrease in the number of NSCs. Meanwhile, in a nucleocytoplasmic separation experiment, we found that NP847 was only expressed in the nucleus and NSCs suffering from OGD or LPS treatment exhibited a decrease in NP847 and Sox2 in the nucleus. Interestingly, the results did not indicate any change in nNOS expression in the nucleus or cytoplasm in OGD-and LPS-treated NSCs. Therefore, based on the above results, we hypothesize that the major reason for NSC renewal is the change in theNP847/Sox2 ratio in the NSC nucleus, not in the expression of nNOS.

Step-wise research has been performed to study which signaling pathways are involved in this process. It has been reported that Shh signaling is central to the CNS for processes such as the self-renewal and development of NSCs along the neuraxis (Tong et al., 2015). The loss of Sox2 exerts an influence on hippocampal development through the loss of Shh. However Sox2-deleted NSCs demonstrate low expression of Shh *in vitro* (Han et al., 2008; Favaro et al., 2009). Recently, Zhang et al. demonstrated that a nNOS-Sox2 complex promoted the transcription of Shh in neurons following glutamate stimulation (Zhang et al., 2016). As shh/Gli has an important role in NSCs, we measured the expression of Shh, which showed an analogous spatio-temporal change in parallel with Sox2. In our experiment, we found that Shh peaked around 3 days after TBI in brain tissue, which was consistent with the changes in NP847 and Sox2 after TBI. Using the OGD model of NSCs, we also investigated whether Shh co-localized with Sox2 and if its expression was down-regulated in a similar manner as Sox2. In addition, when we interfered with the expression of Sox2, the expression of Shh decreased. Based on these results, we demonstrated that the NP847-Sox2 complex enhanced the renewal of NSCs via the Shh/Gli signaling pathway following brain injury. However, it should be mentioned that there are several other signaling pathways related to Sox2 (Liu et al., 2013); therefore, we cannot exclude the possibility that other transcriptional targets of Sox2 may also be involved in this procedure. Thus, this topic requires further research.

Moreover, our studies showed that NP847 and CaMKII partly co-localized in the hippocampus on the third day after TBI, and this result validates the viewpoint that CaMKII phosphorylates nNOS at ser847 after TBI. In addition, we also found that when CaMKII was silenced, the expression of NP847 decreased, which was followed by a decrease in NSC proliferation; this was the same decrease as observed in the OGD and LPS models. Therefore, we presume that CaMKII phosphorylates nNOS at ser847 and then NP847 interacts with Sox2 to enhance neural progenitor/stem cell self-renewal via the promotion of Shh transcription.

**Figure 7:**
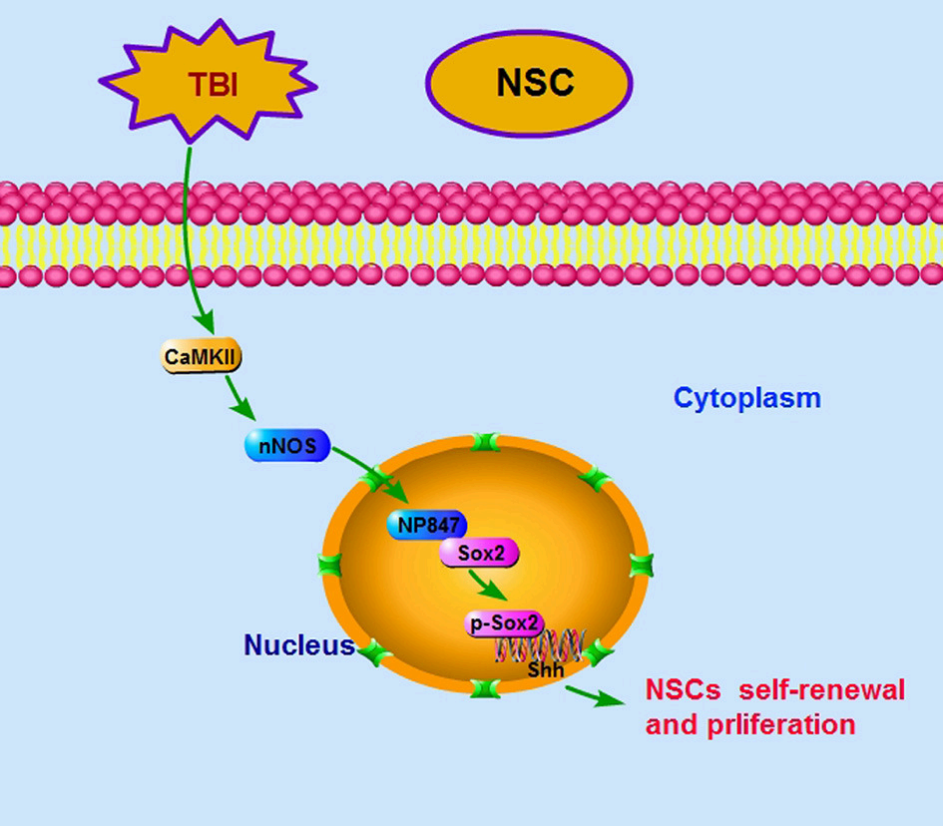
**Ideograph of the NP847-Sox2 complex-mediated enhancement of NSC self-renewal by increasing the expression of Shh**. CaMKII phosphorylates nNOS at ser847, and then the NP847-Sox2 complex enhances the transcription of Shh. CaMKII, calcium-calmodulin-dependent protein kinase II; Ptch1, Patched 1; Smo, smoothened.

Above all, our findings demonstrate that it is NP847 expression in the NSC nucleus, not nNOS, that plays a critical role in the mobilization of NSCs. Our results suggest that CaMKII phosphorylates nNOS to NP847 and this molecule interacts with Sox2 to enhance NSC renewal against brain injury via promotion of the Shh/Gli signaling pathway (Figure 7).

## Author contributions

JG: conception and design, immunoprecipitation, data analysis and interpretation, manuscript writing; YB: cell culture, cell proliferation assay, collection, and assembly of data; JC: conception and design; XZ: technical support; CH: immunofluorescence, collection, and assembly of data; RJ: Western blot analysis; QL: manuscript writing; YL: technical support; XX: conception and design; WS: conception and design, data analysis and interpretation, manuscript writing.

### Conflict of interest statement

The authors declare that the research was conducted in the absence of any commercial or financial relationships that could be construed as a potential conflict of interest.
